# Identification of Resistance Loci to Avian Leukosis via Genome-Wide Association Analysis in Chengkou Mountain Chickens

**DOI:** 10.3390/ani15101365

**Published:** 2025-05-09

**Authors:** Yuhang Li, Min Tan, Guang Yang, Qinwen Xu, Qigui Wang, Haiwei Wang, Xi Lan

**Affiliations:** 1 College of Animal Science, Southwest University, Chongqing 400715, China; lyh1529824967@163.com (Y.L.); 18225473160@163.com (M.T.); yg1551686096@163.com (G.Y.); 18981514003@163.com (Q.X.); 2 Chongqing Academy of Animal Sciences, Chongqing 402460, China; wangqigui@hotmail.com

**Keywords:** ALV, Chengkou mountain chicken, GWAS, resistance loci, tumorigenesis

## Abstract

Avian leukosis (AL), a major vertically transmitted infectious disease, poses a significant challenge to the conservation and industrial development of indigenous chicken breeds in China. In this study, Chengkou mountain chickens were used as a model to systematically identify genetic markers associated with resistance to avian leukosis virus subgroup J (ALV-J) through a genome-wide association study (GWAS). Genomic DNA was extracted from 500 hens at 300 days of age, and cloacal swabs, plasma, and egg white samples were collected to assess the ALV-J infection status. A total of 325 ALV-positive (ALV+) and 175 ALV-negative (ALV−) individuals were identified. Based on 10× whole-genome resequencing and stringent quality control, 12,644,463 high-quality SNPs were obtained. GWAS revealed a significant enrichment of SNPs on chromosome 6 (Chr6), from which 218 SNPs significantly associated with ALV-J resistance and 49 candidate genes were identified. Gene Ontology (GO) and Kyoto Encyclopedia of Genes and Genomes (KEGG) enrichment analyses showed that many of these genes, including *PTPN13*, *TTF2*, *TIAL1*, *DLG2*, *FBXL7*, *CDH5*, and *CDH11*, are involved in tumorigenesis and immunosuppression through the JAK/STAT signaling pathway and cell adhesion molecule pathways. Additionally, candidate genes, such as *ANKH*, *SLC4A7*, and *SLC5A1*, were found to potentially regulate ALV-J infection by modulating membrane transport and inflammatory responses. This study is the first to identify ALV-J resistance-associated genetic markers in Chengkou mountain chickens, revealing key genes related to immune regulation, membrane function, and tumor development. The findings provide a foundational molecular basis for disease-resistant breeding in poultry.

## Simple Summary

Avian leukosis is a vertically transmitted disease that severely impacts the development of indigenous chicken breeds in China. This study aimed to identify genetic resistance factors against avian leukosis virus subgroup J in Chengkou mountain chickens. By analyzing 500 laying hens using whole-genome sequencing, researchers discovered key genes and signaling pathways associated with disease resistance, which may influence immune responses and cellular functions to combat viral infection. These findings provide valuable molecular markers for breeding disease-resistant chickens and offer new insights into the pathogenesis and control strategies of avian leukosis, contributing to sustainable poultry farming.

## 1. Introduction

Avian leukosis (AL) is a viral infectious disease in poultry caused by the avian leukosis virus (ALV), characterized by severe immunosuppression and tumorigenic potential. Livestock production, particularly poultry farming, plays a pivotal role in the socio-economic development of many countries worldwide. Among poultry species, the domestic chicken (Gallus gallus domesticus) stands out due to its short generation interval and adaptability to a wide range of agro-ecological environments, making it one of the most widely distributed avian species globally [1]. As the most extensively raised livestock species, chickens serve as a vital source of high-quality protein and supplemental income for rural households, especially in resource-limited regions. Their popularity is largely attributed to their advantageous traits, including robust disease resistance, adaptability to harsh environments, and efficient utilization of low-quality feed resources [2].

ALV belongs to the C-type retrovirus family and possesses a lipid envelope [3,4]. It is classified into 11 subgroups (A–K) based on the gp85 envelope glycoprotein [5], among which the J subgroup (ALV-J) exhibits the highest pathogenicity [6], with widespread prevalence and substantial economic consequences globally. ALV transmission occurs both vertically and horizontally [7], complicating eradication efforts. Clinically, ALV infection leads to tumor formation, immune suppression, and increased mortality in poultry [8], particularly under intensive farming conditions that facilitate viral spread. Although p27 antigen-based diagnostic assays have been applied in eradication programs, current control strategies remain challenged by the virus’s long incubation period and limitations in detection methods [9].

In recent years, genome-wide association studies (GWASs) have made remarkable contributions to disease-resistance research. By detecting linkage disequilibrium between genetic variants and traits of interest, GWAS enables the identification of significant single nucleotide polymorphisms (SNPs) correlated with phenotypic variation, providing powerful insights into the genetic architecture of disease resistance [10,11,12]. For example, Wossenie Mebratie et al. employed a mixed linear model to identify 11 quantitative trait loci (QTLs) and numerous SNPs associated with body weight and feed conversion efficiency in poultry [13]. Similar GWAS approaches have been applied to traits such as egg quality and disease resistance [14]. In studies of avian influenza resistance, Anna Wolc and colleagues utilized SNP arrays to identify resistance-associated genomic regions, offering key targets for selective breeding, although causal genes remain elusive due to the polygenic nature of the trait [15]. In another study, Xiao et al. identified significantly enriched SNPs on chromosome 5 associated with Salmonella resistance, laying the groundwork for breeding against pullorum disease [16]. Collectively, these findings underscore the utility of GWAS in poultry genetic improvement and disease resistance and provide a valuable reference for future investigations into ALV resistance loci and candidate genes [17,18].

ALV infection is known to reduce egg production, induce tumorigenesis, and suppress the immune system, thereby posing a serious threat to poultry health. Although it is known that ALV enters host cells via specific receptors such as Tva, Tvb, Tvc, and chNHE1, the genetic basis of host resistance remains poorly understood, with limited studies addressing resistance loci and genes associated with ALV-J. The indigenous Chengkou mountain chicken, a local Chinese breed, exhibits strong adaptability and disease resistance. Preliminary long-term AL eradication programs involving multiple generations of birds revealed a significant variation in infection rates among different populations. Notably, some groups exhibited markedly lower infection rates after several rounds of purification, while others showed no obvious improvement, suggesting a potential genetic basis for resistance. In this context, the present study aims to identify resistance-associated loci against ALV-J through whole-genome resequencing and GWAS, thereby providing a genetic foundation for resistance breeding in poultry.

## 2. Materials and Methods

### 2.1. Sample Collection

The experimental population consisted of Chengkou mountain chickens, a native Chinese breed, all raised under identical husbandry conditions. The experimental cohort was established based on preliminary avian leukosis (AL) screening, from which 500 hens—approximately 300 days of age—were randomly selected from three genetic lines: A, R, and D. For each individual, cloacal swabs, egg white, plasma, and whole blood samples were collected according to experimental requirements. In addition, liver, spleen, and kidney tissues were sampled from both ALV-positive and uninfected control groups of 17-week-old hens from the D line, with three biological replicates per group.

### 2.2. Phenotypic Data Collection

Serum samples were tested for ALV-J antibodies using a commercial ELISA kit (IDEXX, Beijing, China), following the manufacturer’s instructions. Plasma, egg white, and cloacal swab samples were analyzed for ALV p27 antigen using an ELISA kit provided by Harbin Gosun Biological Technology Co., Ltd. (Harbin, China), in accordance with the supplied protocol. A sample was considered positive if the OD_630_ nm of the positive control well exceeded 0.20 and the OD_630_ nm of the negative control well was below 0.10. Samples with an S/P value ≥ 0.20 were classified as positive, while those with an S/P value < 0.20 were deemed negative. Upon obtaining preliminary results, cloacal swabs, egg whites, and serum samples were recollected and subjected to repeat p27 antigen ELISA tests to confirm the phenotypic data for sequencing candidates. Individuals were classified as ALV-positive (ALV+) if any one of the three sample types yielded a positive result. Conversely, individuals were defined as ALV-negative (ALV−) only if all three tests returned negative results. For each genetic line, an equal proportion of individuals with no prior record of ALV positivity was selected as the uninfected control group.

### 2.3. Genotypic Data Acquisition

Genomic DNA was extracted using the PCI (phenol–chloroform–isoamyl alcohol) method [19], and the DNA quality was assessed using the Agilent 5400 system (Agilent, Wilmington, DE, USA). Sequencing libraries were prepared using the TruSeq Nano DNA HT sample preparation kit (Illumina, San Diego, CA, USA), with unique index sequences incorporated into each sample fragment. The libraries were sequenced on the Illumina NovaSeq 6000 platform, generating 150 bp paired-end reads with an average insert size of approximately 350 bp and a sequencing depth of 10×.

### 2.4. Data Processing

#### 2.4.1. Quality Control

Raw sequencing reads were filtered based on the following criteria: (1) removal of reads with ≥10% unidentified nucleotides (N); (2) removal of reads with >50% low-quality bases (Phred score < 5); (3) removal of reads with adapter sequence alignment ≥10 nucleotides, allowing ≤10% mismatches; (4) removal of duplicate sequences resulting from PCR amplification, defined as read pairs with identical sequences.

#### 2.4.2. Alignment to the Reference Genome

Filtered high-quality reads were aligned to the chicken reference genome (bGalGal1.mat.broiler.GRCg7b, from the NCBI database) using the BWA software (version 0.7.8). Duplicate reads were removed using SAMTOOLS (version 0.1.19) [20].

#### 2.4.3. Variant Calling and Annotation

SNPs were identified using GATK. The resulting variants were filtered with Plink (version 1.07) and Vcftools (version 0.1.15) using the following thresholds: QD < 2.0, MQ < 40.0, FS > 60.0, SOR > 3.0, MQRankSum < –12.5, and ReadPosRankSum < –8.0. Vcftools was further used to retain high-quality SNPs with parameters --max-missing 0.9 and --maf 0.05. SNP annotation was conducted using ANNOVAR (HG19).

#### 2.4.4. Phylogenetic Tree Analysis

Following SNP detection, the genetic distance between individuals was calculated using the high-quality filtered SNPs. A distance matrix was generated using Treebest (version 1.9.2), and a phylogenetic tree was constructed based on the neighbor-joining method [21].

#### 2.4.5. Principal Component Analysis (PCA)

PCA was performed using the GCTA software package (version 1.24.2) [22] based solely on autosomal SNP data from n individuals. Multiallelic sites (more than two alleles) and mismatched data were excluded. Each SNP at position (i, k) was encoded as 0 (homozygous for the reference allele), 1 (heterozygous), or 2 (homozygous for the alternative allele). A standardized genotype matrix M (n × S) was calculated as follows:

$$d_{ik}^{\prime }=\frac{\left(d_{ik}-E\left(d_{k}\right)\right)}{\sqrt{E\left(d_{k}\right)\times (1-\frac{E\left(d_{k}\right)}{2})/2}}$$

where E(d_k) is the average genotype at SNP k. The sample covariance matrix X (n × n) was calculated using X = MMᵀ/S.

#### 2.4.6. Population Structure Analysis

The population structure was analyzed using PLINK, and the genetic structure and lineage information were further inferred using sNMF, frappe, or Admixture (version 1.3.0). The ancestral proportions were estimated by integrating sparse non-negative matrix factorization (sNMF) with least-squares optimization. The optimal number of ancestral populations was determined at the first inflection point of the cross-entropy curve. This estimate was then validated by comparing the results from the phylogenetic tree and PCA.

#### 2.4.7. Genome-Wide Association Study

Fisher’s exact test was applied to evaluate the association between two categorical variables using 2 × 2 contingency tables. For the GWAS analysis, GEMMA (version 0.94.1) was used to compute the kinship matrix (K), while GCTA (version 1.24.2) was used to estimate the population structure (Q) [23,24,25].

#### 2.4.8. Result Visualization

To determine the significance threshold for *p*-values, a Bonferroni correction was applied, with a *p*-value threshold of 5 × 10⁻⁶ set to identify SNPs significantly associated with the trait. Here, n represents the total number of SNPs analyzed, and chr denotes the number of chromosomes.

$$p\text{-}value=-\log10\left(\frac{1}{\left(n∕\mathrm{ch}r\right)}\right)$$

Q–Q plots and Manhattan plots were generated using the CMplot package.

#### 2.4.9. GO and KEGG Enrichment Analyses

GO functional enrichment and KEGG pathway analyses were performed on SNPs identified above the significance threshold in the genome-wide association study using the KOBAS online platform, based on the Gene Ontology (GO) database (http://geneontology.org) and the Kyoto Encyclopedia of Genes and Genomes (KEGG) database (http://www.kegg.jp/kegg, accessed on 27 March 2025) [26,27]. GO terms and KEGG pathways with adjusted *p*-values < 0.05 were considered statistically significant.

### 2.5. RNA-Seq Analysis

#### 2.5.1. Sample Collection

Tissue samples were rinsed with phosphate-buffered saline (PBS) and trimmed into small pieces before being immersed in RNAlater stabilization solution. After incubation at room temperature, the RNAlater solution was discarded, and only the tissue specimens were retained. The samples were then stored at −80 °C until further use. Subsequently, the preserved tissues were sent to Novogene Co., Ltd. (Beijing, China) for transcriptome sequencing.

#### 2.5.2. Data Processing

Low-quality and contaminant reads were removed from the raw FASTQ data using FastQC to obtain clean reads. These clean reads were then aligned to the chicken reference genome (GCF_016699485.2) using HISAT2 (version 2.1.0), and only reads that successfully mapped to the genome were retained for downstream analysis [28,29]. The sample correlation was assessed using Pearson correlation coefficients and a principal component analysis (PCA). Gene expression levels were quantified using HTSeq (version 0.6.0), and differential expression analysis was performed with DESeq2. The genes were considered differentially expressed if they met the criteria of *p* ≤ 0.05 and |log_2_(fold change)| ≥ 1 [30].

### 2.6. Quantitative Real-Time PCR (qRT-PCR)

Three candidate genes were randomly selected for quantitative real-time PCR (qRT-PCR) validation. The reactions were performed using a SYBR Premix Ex Taq II kit (Takara, Japan). The thermal cycling conditions were as follows: initial denaturation at 95 °C for 2 min; followed by 40 cycles of 95 °C for 5 s and 60 °C for 30 s; and a melting curve analysis consisting of 95 °C for 10 s, 65 °C for 5 s, and 95 °C for 5 s. The gene expression levels were calculated using the 2^−ΔΔCT^ method based on threshold cycle (CT) values. A statistical analysis and bar graph visualization were performed using the GraphPad Prism software (Bioc 3.15). Differences with *p* < 0.05 or *p* < 0.01 were considered statistically significant [31].

## 3. Results

### 3.1. Genotypic and Phenotypic Data

#### Phenotypic Data

In this study, families with significant differences in ALV infection status were selected from populations that had undergone multiple generations of avian leukosis purification. A total of 1050 chickens were sampled for cloacal swabs, egg white, and serum. Using ELISA to detect the ALV p27 antigen, 500 individuals were selected for whole-genome resequencing, comprising 325 ALV+ and 175 ALV– individuals, yielding an ALV–/ALV+ ratio of approximately 2:1 (Table A1).

### 3.2. SNP Detection

#### 3.2.1. Alignment to the Reference Genome

Genomic DNA was extracted from a total of 500 chickens and subjected to whole-genome sequencing. The DNA quality assessment results are presented in Table A2. The chicken reference genome size was 1,049,948,333 base pairs. The alignment rate of the population samples ranged from 98.44% to 99.64%, with an average sequencing depth of 12.08× and an average genome coverage of 98.50%.

#### 3.2.2. SNP Detection and Annotation

A total of 20,959,220 raw SNPs were initially detected, and after filtering, 12,644,463 high-quality SNPs were retained. A remarkable accumulation of SNPs was observed around the 5 Mb region on chromosome 6 (Chr6). Annotation revealed that most of the filtered SNPs were enriched in intronic and intergenic regions, while a smaller proportion were located in upstream, exonic, and downstream regions of genes. A further analysis showed that the majority of SNP variations were caused by base transitions (Table A3).

### 3.3. Population Genetic Diversity Analysis

**Figure 1:**
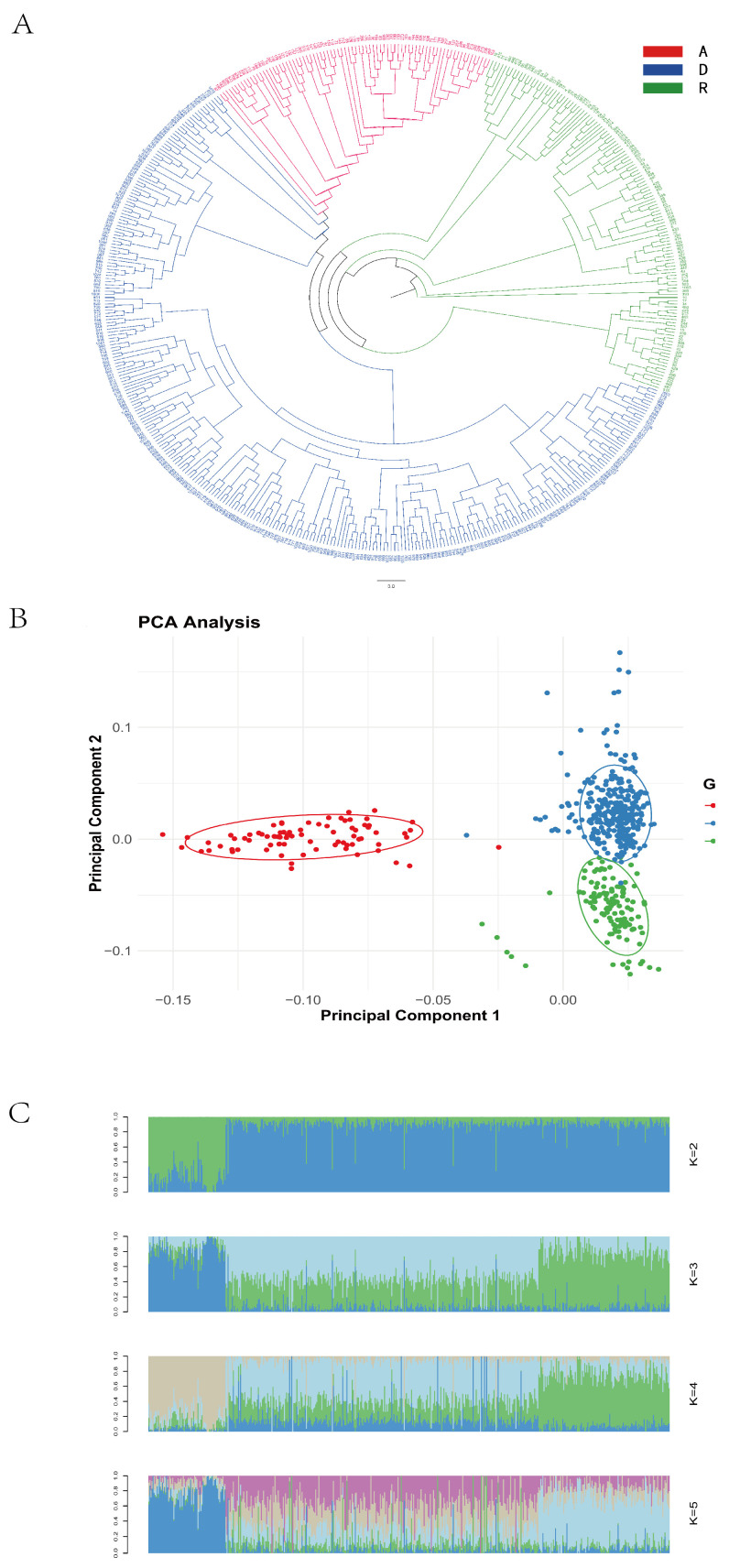
Population genetic diversity. (**A**) Phylogenetic tree; (**B**) principal component analysis plot; (**C**) population genetic structure plot.

The phylogenetic tree revealed clear clustering patterns consistent with the line-specific classifications (Lines A, R, and D), as recorded during the sample collection (Figure 1A). A principal component analysis (PCA) showed that some individuals from Line D clustered with Lines A and R, while, overall, the three lines demonstrated good intra-line clustering and clear inter-line separation (Figure 1B). When the number of ancestral populations was set to three, each individual’s genome exhibited clear structural grouping (Figure 1C), indicating that the 500 resequenced samples originated from three ancestral populations.

Based on the results of the population diversity analyses, the sequenced samples were determined to originate from three ancestral populations. This finding is consistent with the three lines recorded during sample collection, confirming the accuracy of population classification and validating the feasibility of conducting a line-specific genome-wide association analysis in the subsequent steps.

### 3.4. Genome-Wide Association Analysis

**Figure 2:**
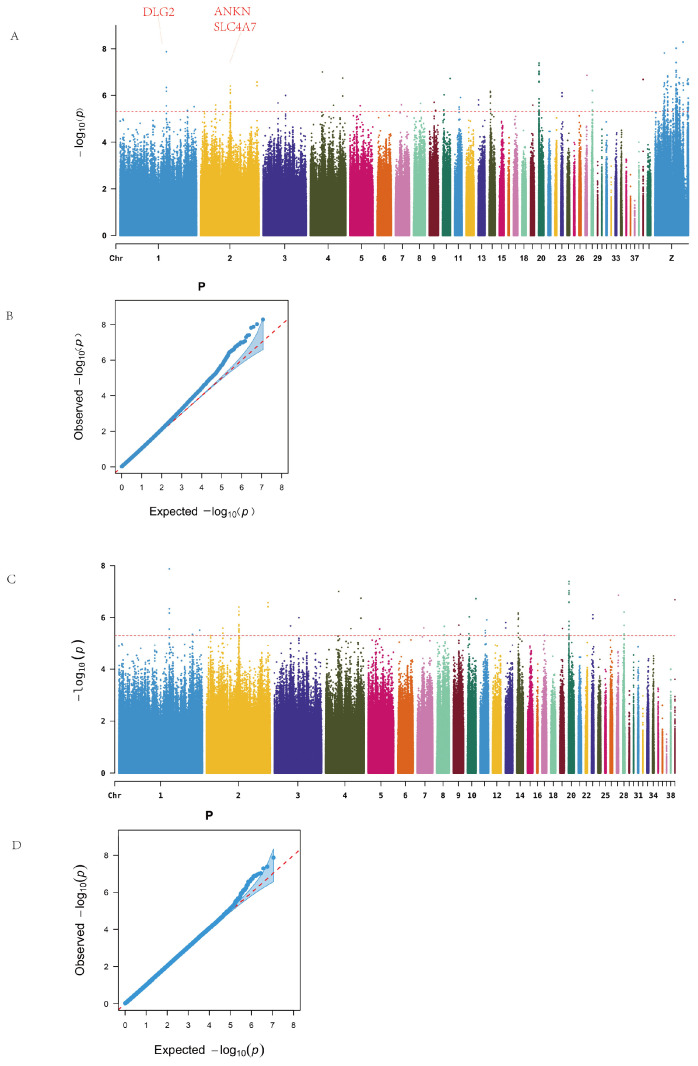
Manhattan and Q–Q plots. (**A**) Overall Manhattan. (**B**) Overall Q–Q. (**C**) Excluding sex chromosomes Manhattan. (**D**) Excluding sex chromosomes Q–Q.

Genome-wide association analysis revealed significant loci on chromosomes 1, 2, 4, 14, 20, 28, and the Z chromosome, as illustrated in the Manhattan plot (Figure 2A). No continuous regions of significance were observed on the Z sex chromosome. A total of 218 SNPs surpassing the genome-wide significance threshold were identified and mapped to 49 candidate genes. Functional annotations showed that two SNPs were located in exonic regions, including one synonymous mutation and one non-synonymous mutation (on chromosome Z). Additionally, 76 SNPs were located in intergenic regions (Table A4). Table 1 lists the top 20 SNPs and their corresponding genes, including a synonymous SNP on chromosome 1 with the most significant *p*-value.

Among the significantly enriched genes, the Z chromosome harbored the largest number of SNPs, with BNC2 and NRG1 annotated with 15 and 13 SNPs, respectively. On the autosomes, ANKH on chromosome 2 and EIF6 on chromosome 20 exhibited substantial SNP enrichment. Additionally, genes such as FBXL7 and SLC4A7 were also significantly enriched (Table A5).

Since no continuous high-signal SNP regions were observed on the Z chromosome, and to avoid the dilution of potential association signals by the high mutation load on the sex chromosome, we excluded the Z chromosome data from the subsequent analyses. This adjustment also aimed to better explore intra- and inter-line differences among chicken populations. Therefore, follow-up genome-wide association analyses for ALV resistance were conducted both within individual lines and across the entire population to further identify potential associated loci and candidate genes.

After removing sex chromosome data, the resulting Manhattan plot revealed continuous high-signal regions on chromosomes 1, 2, and 20 (Figure 2B). A total of 78 significant SNPs were identified, spanning 17 intergenic regions and 21 genes, including one synonymous mutation located in an exon. Notable candidate genes included ANKH (12 associated SNPs), EIF6 (11 SNPs), and SLC4A7, DLG2, and FBXL7, which are widely implicated in cellular inflammation, immune response, and tumor development.

**Figure 3:**
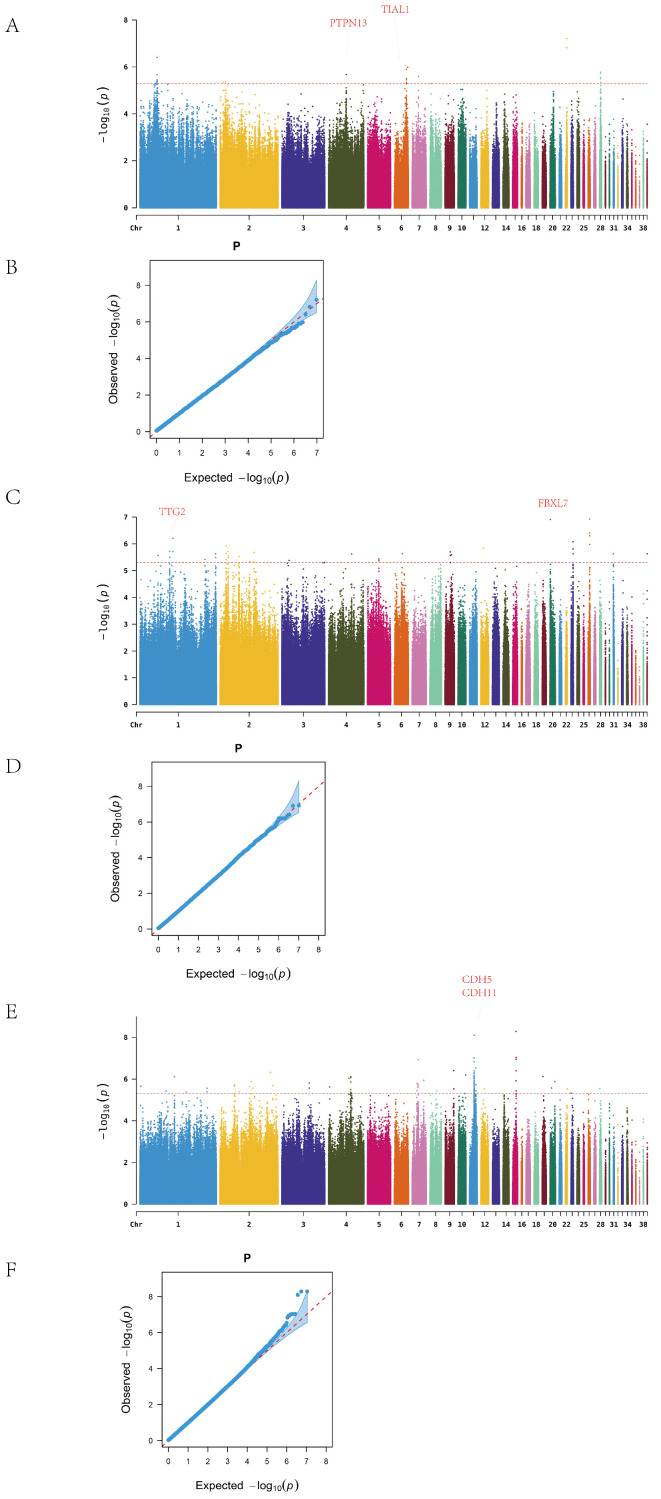
Manhattan and Q–Q plots. (**A**) A series Manhattan. (**B**) A series Q–Q. (**C**) R series Manhattan. (**D**) R series Q–Q. (**E**) D series Manhattan. (**F**) D series Q–Q.

Further within-line analysis revealed the following findings:

In Line A, a total of 26 ALV-negative and 48 ALV-positive individuals were analyzed. After excluding SNPs on the sex chromosome, genome-wide association analysis identified continuous clusters of significant variants on chromosomes 1, 4, 6, and 28 (Figure 3A). In total, 34 significant SNPs were detected, mapped to 12 candidate genes. The most significant SNP on chromosome 22 was located in an intergenic region, while 11 SNPs on chromosome 1 were concentrated in a single intergenic region. Twelve SNPs were located within gene regions, corresponding to nine genes, among which PTPN13 and TIAL1 are associated with cancer and immune function (Table A6).

In Line R, after excluding sex chromosome data, the Manhattan plot revealed significant SNPs on chromosomes 1, 2, 9, 23, and 26 (Figure 3B). A total of 46 SNPs were identified, distributed across 13 chromosomes and mapped to 20 candidate genes. The most significant SNP on chromosome 26 was located between NGF and FANCE; NGF is known to be involved in nerve growth, while FANCE plays a key role in the Fanconi anemia pathway. On chromosome 1, 14 SNPs were detected, with 7 mapping to the TTF2 gene, which is associated with thyroid development.

In Line D, 92 significant SNPs were identified across 16 chromosomes after removal of sex-linked loci, and these were mapped to 21 candidate genes. High-density SNP regions were detected on chromosomes 1, 4, 7, 11, and 15 (Figure 3C). The most significant SNP on chromosome 15 was located in the YWHAH gene, which has been implicated in viral encephalitis and tumor progression. On chromosome 11, 30 SNPs were concentrated in the intergenic region between CDH5 and CDH11, both members of the cadherin family, which are involved in cancer and vascular disease. In addition, the identification of SLC5A1, a member of the solute carrier (SLC) family, supports the relevance of this gene family in ALV resistance, consistent with previous findings related to SLC4A7.

Q–Q plots confirmed that the observed *p*-value distribution closely followed the expected uniform distribution, indicating no systematic bias in the GWAS results.

### 3.5. GO and KEGG Enrichment Analyses

To comprehensively evaluate the functional roles of candidate genes annotated from significant SNPs, Gene Ontology (GO) and Kyoto Encyclopedia of Genes and Genomes (KEGG) enrichment analyses were performed under the threshold of *p* < 0.05.

**Figure 4:**
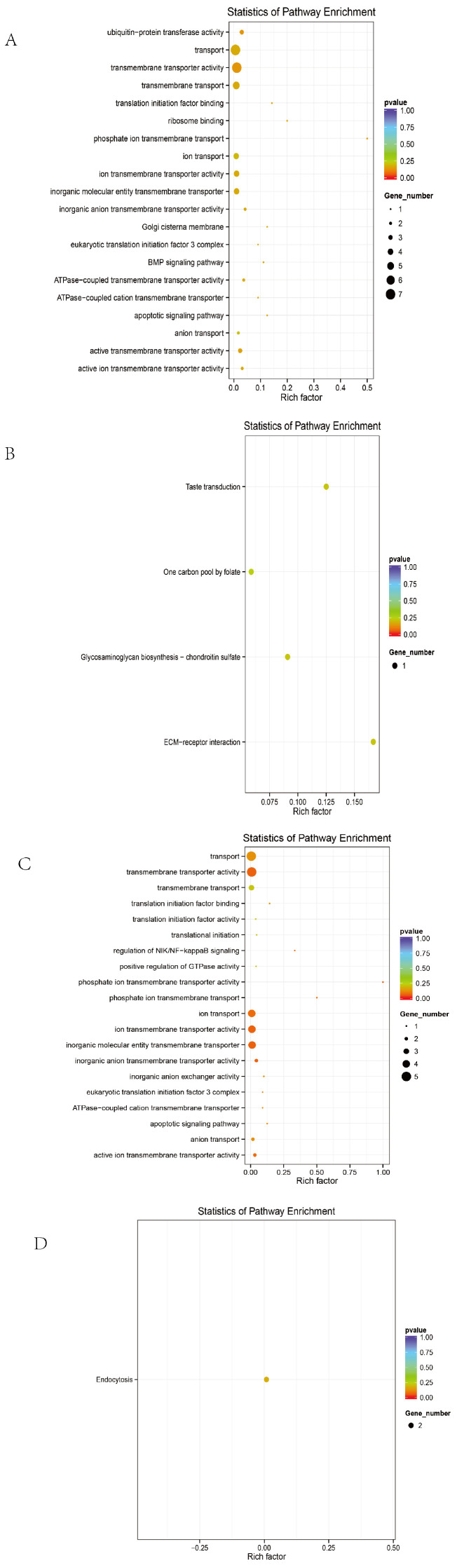
Overall GO and KEGG plots. (**A**) GO map of the entire group without excluding sex chromosomes. (**B**) KEGG map of the entire group without excluding sex chromosomes. (**C**) GO map of the entire group after excluding sex chromosomes. (**D**) KEGG map of the entire group after excluding sex chromosomes.

Before excluding sex chromosomes, the enriched terms included 9 biological processes, 3 cellular components, 15 molecular functions, and 4 KEGG pathways (Figure 4A,B). The top 20 GO terms with the highest enrichment scores are shown in the figure.

After excluding sex chromosomes, an enrichment analysis revealed 16 biological processes, 1 cellular component, 14 molecular functions, and 9 KEGG pathways (Figure 4C,D).

**Figure 5:**
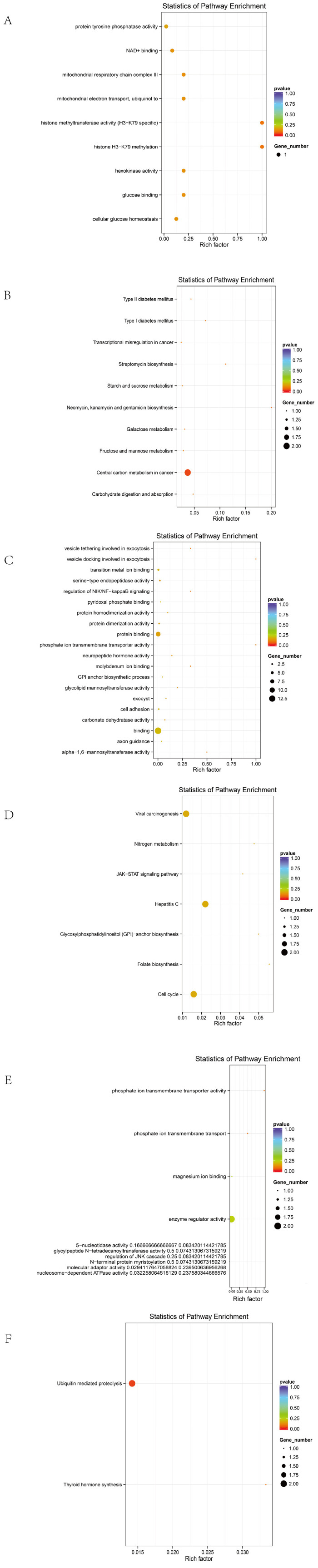
GO and KEGG maps of different strains. (**A**) GO diagram of series A. (**B**) A KEGG diagram. (**C**) R system GO diagram. (**D**) R series KEGG diagram. (**E**) D GO diagram. (**F**) D series KEGG diagram.

Line-specific analyses yielded the following results:

In Line A, 26 biological processes, 10 cellular components, 16 molecular functions, and 10 KEGG pathways were enriched (Figure 5A,B).

In Line R, 25 biological processes, 10 molecular functions, and 2 KEGG pathways were identified (Figure 5C,D).

In Line D, 39 biological processes, 3 cellular components, 22 molecular functions, and 7 KEGG pathways were significantly enriched (Figure 5E,F).

Candidate genes were significantly enriched in membrane-associated signaling pathways and tumorigenesis-related mechanisms, including membrane transporter activity, transmembrane transport, cellular metabolism, endoplasmic reticulum function, ion transport, and cancer-related functions. Representative genes included SLC4A7, SLC5A1, ANKH, EIF6, DLG2, FBXL7, and CDH5.

A KEGG analysis further indicated that these genes are involved in the JAK/STAT signaling pathway, ECM–receptor interaction, glycosaminoglycan biosynthesis, folate-mediated one-carbon metabolism, and other pathways, which are potentially associated with the tumorigenic mechanisms in ALV-infected individuals.

### 3.6. Identification of Candidate Genes by RNA-Seq Analysis

To validate the potential functions of candidate genes identified through GWAS, we integrated RNA-Seq data and performed an intersection analysis between differentially expressed genes and a randomly selected subset of three GWAS candidate genes (ANKH, CDH11, and SLC5A1). All three genes exhibited significant differential expression in the RNA-Seq analysis (*p* < 0.05) (Table A7).

### 3.7. qRT-PCR Validation of Candidate Gene Expression

**Figure 6:**
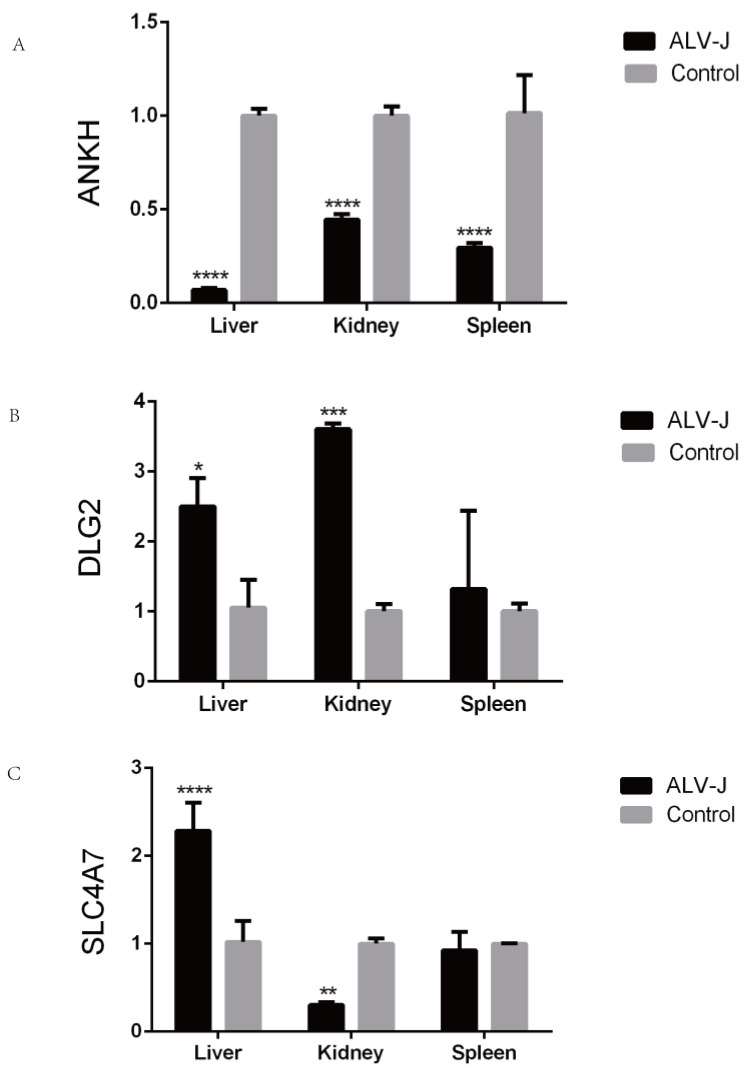
qPCR analysis verifies the expression of candidate genes. (**A**) Expression of ANKH in different tissues. (**B**) Expression of DLG2 in different tissues. (**C**) Expression of SLC4A7 in different tissues. *: *p* < 0.05, **: *p* < 0.01, ***: *p* < 0.001, ****: *p* < 0.0001.

Three candidate genes (ANKH, DLG2, and SLC4A7) were randomly selected for qRT-PCR validation (Figure 6). Under ALV-J infection, ANKH exhibited significant differential expression in the liver, spleen, and kidney tissues. Both DLG2 and SLC4A7 showed significant changes in expression in the liver and kidney tissues.

## 4. Discussion

Avian leukosis virus subgroup J (ALV-J) is a highly contagious disease in poultry, characterized by rapid transmission and the lack of effective vaccines, resulting in substantial economic losses to the poultry industry. Although continuous screening and culling strategies have helped to control the spread of ALV-J, these approaches have not completely eliminated the virus from poultry populations. In 2006, researchers identified the sodium–hydrogen exchanger 1 (NHE1) as a cellular receptor for ALV-J [32]. The study demonstrated that quails and certain wild birds exhibited resistance to ALV-J, while turkeys and some domestic chicken breeds were susceptible. However, the specific genetic loci associated with ALV-J resistance remain largely unknown. In this study, a genome-wide association study (GWAS) was used to identify genetic variants associated with ALV-J susceptibility, providing insights into the host’s genetic determinants of infection.

GWAS revealed several loci significantly associated with ALV-J infection, particularly within genes related to immune response, cancer progression, and membrane function. Among these, *PTPN13*, a known tumor suppressor, showed a strong association with ALV-J susceptibility. *PTPN13* is involved in regulating cell death and migration, and its dysregulation in various cancers suggests that it may influence ALV-J infection by modulating immune responses and intracellular signaling pathways [33,34,35].

Another notable gene, *TTF2*, a thyroid-specific transcription factor, has been implicated in the pathogenesis of multiple cancers, including thyroid carcinoma [36,37,38]. ALV integrates into the host genome and can activate or disrupt proto-oncogene expression, leading to abnormal cell proliferation such as lymphoma. Given ALV’s reliance on host transcriptional machinery, *TTF2* may affect the efficiency of viral replication or integration, thereby influencing susceptibility to ALV-J.

TIAL1, a gene involved in RNA splicing and DNA repair, plays a critical role in B cell development. Mutations in *TIAL1* may impair B cell maturation, contributing to immune tolerance and persistent ALV-J infection [39]. In addition, genes such as *DLG2* [40,41,42] and *FBXL7* [43] are involved in tumor progression and immune regulation. ALV-J infection has been shown to suppress JAK-STAT signaling by upregulating SOCS3 (suppressor of cytokine signaling 3), thereby inhibiting innate immune responses and promoting viral replication [44]. The enrichment of these genes in the JAK-STAT pathway suggests that they may influence ALV-J susceptibility by modulating host immunity.

As ALV-J initiates infection by binding to specific receptors on the host cell membrane, genes involved in membrane structure and function are critical for viral entry and propagation. In this study, several candidate genes associated with membrane function were identified, including *ANKH*, *SLC4A7*, *SLC5A1*, and members of the cadherin (*CDH*) family. These genes are essential for ion transport, cell adhesion, and maintenance of cellular homeostasis—processes that are vital for viral infection.

*ANKH* encodes a transmembrane protein responsible for inorganic pyrophosphate transport [45]. Mutations in *ANKH* have been linked to ion transport disorders and inflammatory conditions, such as arthritis [46,47]. Our findings suggest that *ANKH* may alter ionic homeostasis at the cell surface, thereby influencing ALV-J binding and entry.

*SLC4A7* and *SLC5A1*, members of the solute carrier family, are involved in bicarbonate and glucose transport, respectively. Enrichment of these genes in pathways related to ion transport and cancer metabolism suggests that their dysfunction may alter membrane permeability, facilitating viral invasion and disease progression [48,49,50,51,52,53,54,55,56,57]. Furthermore, their association with cancer-related pathways supports the notion that changes in membrane function influence both viral infection and tumorigenesis.

The cadherin family genes *CDH5* and *CDH11* are crucial for cell–cell adhesion and tissue integrity. These proteins are known to participate in tumor immune responses and can enhance antitumor immunity. Notably, *CDH5* and *CDH11* were enriched in the JAK-STAT signaling pathway, which has been associated with immune regulation. High expression of *CDH5* can enhance CD8^+^ T cell activity, thereby suppressing tumor growth. Their enrichment in this pathway suggests a role in modulating host susceptibility to ALV-J by influencing immune signaling [58,59,60].

In addition to identifying candidate genes, this study highlights several key biological pathways involved in ALV-J infection and immune suppression. The JAK-STAT signaling pathway, crucial for immune cell signal transduction, was significantly enriched. This pathway has been linked to various immune disorders and cancers [61,62,63,64]. In chicken macrophages and DF-1 cells, ALV-J infection has been shown to reduce STAT1 phosphorylation, impairing interferon responses and facilitating immune evasion [65]. Thus, ALV-J may promote tumorigenesis by disrupting JAK-STAT signaling and weakening host antiviral defenses.

Enrichment of the extracellular matrix (ECM)–receptor interaction pathway was also observed. This pathway plays a pivotal role in cell adhesion, migration, and proliferation during tumor development [66], and its involvement in cancers such as prostate and gastric cancer is well-documented [67,68,69]. ALV may disrupt ECM-receptor interactions, thereby promoting viral entry, immune evasion, and tumorigenesis. Additional enriched pathways, including glycosaminoglycan biosynthesis and folate-mediated one-carbon metabolism, are involved in cell proliferation, migration, and survival—processes often dysregulated in cancer [70,71,72,73,74].

Although this study successfully identified potential loci associated with ALV-J resistance in Chengkou mountain chickens via GWAS, several limitations should be acknowledged. Firstly, despite applying Bonferroni correction, false positives may still arise due to multiple testing and residual population stratification. Secondly, phenotypic misclassification may affect the strength of association signals. While RNA-Seq and qPCR validations were partially conducted, further functional genomics studies and cross-population validations are necessary to confirm the biological relevance of these candidate loci.

## 5. Conclusions

This study identified multiple candidate genes and signaling pathways associated with immune regulation, cell membrane function, and tumorigenesis through a genome-wide association analysis. Genetic variations in these genes may underlie differences in susceptibility to ALV-J infection among various chicken lines. However, the precise mechanisms by which these genes influence ALV-J infection and immunosuppression require further investigation. Future research will focus on functional studies of these candidate genes—particularly gene knockout and overexpression experiments—to elucidate their specific roles in ALV-J pathogenesis.

In addition, the application of these genetic markers in selective breeding programs for ALV-J-resistant poultry should be considered a long-term strategy for disease control. Nonetheless, challenges such as underdeveloped breeding systems and limited funding for local chicken breeds remain significant barriers to implementation. Overall, this study provides new insights into the genetic susceptibility of poultry to ALV-J infection and lays a foundational framework for future disease-resistance breeding efforts.

## Figures and Tables

**Table 1:** The top 20 *p*-values of SNPs.

Chr	Num-snp	LOG10 (*p*-Value)	Peak-Effect	Gene
Chr1	1	8.87361	exon, synonymous	LOC107049245
ChrZ	3	8.28455	intronic	DTWD2
ChrZ	13	8.02251	intronic	NRG1
Chr1	3	7.87361	intergenic	
ChrZ	9	7.81761	intronic	ANKDD1B
Chr20	11	7.38612	intergenic	EIF6
Chr20	3	7.29405	intronic	FAM83C
Chr4	1	7.00458	intergenic	
ChrZ	3	6.87857	intergenic	
Chr27	1	6.86258	intronic	HAP1
ChrZ	15	6.82981	intronic	BNC2
ChrZ	5	6.75517	intergenic	
Chr4	1	6.74306	intergenic	
Chr10	1	6.72638	intronic	UACA
ChrZ	1	6.69983	intergenic	
Chr39	1	6.68551	intronic	SCYL1
ChrZ	3	6.62974	intronic	TRIM36
Chr2	2	6.57298	intronic	TRAPPC9
ChrZ	2	6.55467	intergenic	
ChrZ	1	6.5532	intergenic	

## Data Availability

The whole-genome sequencing data generated in this study are available in the NCBI database under accession number PRJNA1242535.

## Appendix A

**Table A1:** Sample phenotype grouping.

Strains	Quantity	Positivity Rate
A	74	64.86%
R	126	65.08%
D	300	65.00%
The whole group	500	65.00%

**Table A2:** DNA quality testing.

Sample Number	Concentration (ng/μL)	Volume (μL)	Total Volume (μg)
2	333.92	60	20.0352
4	323.06	60	19.38384
5	92.96	60	5.5776
6	62.35	60	3.74076
7	254.11	60	15.24678
8	39.93	60	2.39562
10	82.13	60	4.92768
12	72.61	60	4.35636
13	137.45	60	8.24706
14	185.33	60	11.12004
15	84.86	60	5.09166
17	57.82	60	3.46932
18	383.7	60	23.02212
19	105.67	60	6.33996
21	48.39	60	2.9031
23	242.63	60	14.55762
24	343.41	60	20.60436
26	426.85	60	25.61112
27	102.87	60	6.17226
29	102.04	60	6.12258
33	173.82	60	10.4292
34	393.56	60	23.61348
36	441.25	60	26.47494
39	410	60	24.6
40	145.3	60	8.71794
41	251.94	60	15.11664
42	301.73	60	18.10368
43	169.51	60	10.1703
44	158.16	60	9.48972
48	88.47	60	5.30814
49	388.93	60	23.33592
51	453.35	60	27.20106
53	171.04	60	10.26264
54	96.82	60	5.80944
55	213.54	60	12.81264
56	190.93	60	11.45568
58	150.67	60	9.0402
59	356.21	60	21.37248
62	72.28	60	4.33698
63	71.15	60	4.26912
64	111.14	60	6.66816
65	119.59	60	7.17552
70	115.36	60	6.92154
72	108.66	60	6.51954
74	373.25	60	22.395
76	143.62	60	8.61714
78	189.07	60	11.3439
83	130.06	60	7.80372
85	426	60	25.56
87	676	60	40.56
91	87.84	60	5.27028
94	449.58	60	26.97504
95	580	60	34.8
96	57.9	60	3.47388
98	197.61	60	11.85642
99	450	60	27
103	145.95	60	8.7567
104	125.93	60	7.5558
105	448.76	60	26.92566
108	688	60	41.28
109	194.82	60	11.68944
110	238.42	60	14.3052
113	176.43	60	10.58556
118	53.21	60	3.19272
119	110.32	60	6.61896
121	48.26	60	2.8953
122	74.11	60	4.44636
125	8.6	60	0.51612
130	179.7	60	10.78212
131	105.82	60	6.34932
132	416	60	24.96
136	95.84	60	5.7501
138	236.14	60	14.16858
140	312.09	60	18.72558
144	638	60	38.28
145	59.33	60	3.55956
147	53.13	60	3.18756
150	65.92	60	3.9552
152	92.91	60	5.5743
153	120.31	60	7.21854
157	86.46	60	5.18742
158	395.69	60	23.7414
159	327.93	60	19.67598
164	249.29	60	14.95746
170	95.15	60	5.70894
171	51.24	60	3.07452
174	131.11	60	7.86636
175	70.19	60	4.21158
176	135.27	60	8.11626
178	132.02	60	7.92138
988	914	60	54.84
989	806	60	48.36
990	906	60	54.36
992	864	60	51.84
993	738	60	44.28
995	1080	60	64.8
996	734	60	44.04
997	812	60	48.72
998	746	60	44.76
999	1180	60	70.8
1002	914	60	54.84
1003	578	60	34.68
1006	712	60	42.72
1007	742	60	44.52
1009	956	60	57.36
1014	734	60	44.04
1017	872	60	52.32
1018	774	60	46.44
1022	912	60	54.72
1026	1040	60	62.4
1027	906	60	54.36
1028	950	60	57
1029	704	60	42.24
1030	832	60	49.92
1031	626	60	37.56
1032	648	60	38.88
1033	936	60	56.16
1035	856	60	51.36
1036	788	60	47.28
1037	916	60	54.96
1038	1056.57	60	63.39444
1041	668	60	40.08
1042	484	60	29.04
1043	752	60	45.12
1044	624	60	37.44
1045	706	60	42.36
1046	760	60	45.6
1047	764	60	45.84
1048	554	60	33.24
180	130.45	60	7.82676
182	559.31	60	33.55836
183	264.75	60	15.88494
188	237.23	60	14.23362
194	406	60	24.36
197	396.05	60	23.76318
199	24.28	60	1.45656
202	231.18	60	13.87086
207	81.14	60	4.8681
215	56.76	60	3.40578
219	308.82	60	18.52908
228	175.81	60	10.54872
239	95.67	60	5.74038
245	34.44	60	2.06658
246	67.65	60	4.05888
252	106.88	60	6.4125
257	89.11	60	5.34648
260	43.49	60	2.6094
266	53.42	60	3.20538
273	264.17	60	15.85026
275	247.17	60	14.83044
285	86.55	60	5.19282
297	95.03	60	5.70168
303	123.18	60	7.39068
308	46.62	60	2.7969
313	41.99	60	2.51922
318	40.32	60	2.4189
319	71.24	60	4.2744
320	161.38	60	9.68268
322	90.1	60	5.40618
477	445.09	60	26.70522
480	60.41	60	3.62472
481	73.05	60	4.38288
482	83	60	4.98018
483	106.92	60	6.41502
484	153.53	60	9.21156
491	76.04	60	4.5624
492	44.66	60	2.67972
493	56.68	60	3.4008
494	252.67	60	15.1602
496	88.11	60	5.28672
498	51.33	60	3.07992
501	64.2	60	3.85224
503	222.73	60	13.36356
507	62.04	60	3.72216
508	54.51	60	3.27042
511	43.16	60	2.5893
512	264.55	60	15.87306
516	44.7	60	2.68218
517	49.93	60	2.99598
522	52.72	60	3.16326
523	74.03	60	4.44156
526	71.29	60	4.2774
527	38.27	60	2.29602
536	65.01	60	3.9006
537	358.86	60	21.53172
538	57.96	60	3.4773
539	153.7	60	9.22188
540	37.69	60	2.26122
542	64.75	60	3.88476
543	117.92	60	7.07538
544	140.3	60	8.4177
545	127.35	60	7.64094
548	77.15	60	4.62882
549	118.49	60	7.10922
551	66.21	60	3.97272
558	90.19	60	5.41134
559	356.5	60	21.38988
567	250.12	60	15.00696
568	198.34	60	11.9004
569	162.51	60	9.75066
571	142.54	60	8.55252
572	634	60	38.04
573	536	60	32.16
574	694	60	41.64
575	630	60	37.8
579	514	60	30.84
586	622	60	37.32
588	632	60	37.92
590	620	60	37.2
591	712	60	42.72
592	608	60	36.48
594	562	60	33.72
595	752	60	45.12
598	674	60	40.44
599	732	60	43.92
600	626	60	37.56
601	868	60	52.08
602	784	60	47.04
604	395.93	60	23.7558
719	488.21	60	29.2923
723	530	60	31.8
724	728	60	43.68
725	530	60	31.8
726	800	60	48
324	352.69	60	21.16158
332	334.49	60	20.06934
334	203.39	60	12.20364
335	200.28	60	12.0165
336	181.36	60	10.88142
337	400	60	24
344	307.96	60	18.47772
345	149.27	60	8.95602
347	360	60	21.6
348	316	60	18.96
352	197.95	60	11.8767
358	485.43	60	29.1255
360	616	60	36.96
361	704	60	42.24
362	219.06	60	13.14342
364	21.09	60	1.26558
369	282.26	60	16.93548
370	464	60	27.84
372	538	60	32.28
374	508	60	30.48
375	57.33	60	3.43962
377	738	60	44.28
381	266.58	60	15.99456
386	356.41	60	21.38448
387	435	60	26.1
393	25.58	60	1.53456
395	397.42	60	23.84514
396	450	60	27
405	700	60	42
407	790	60	47.4
408	808	60	48.48
409	646	60	38.76
412	1.98	60	0.1188
416	336	60	20.16
423	392	60	23.52
424	379.03	60	22.74198
426	32.25	60	1.935
727	24.89	60	1.4931
728	475	60	28.5
729	326.1	60	19.56594
730	590	60	35.4
737	44.43	60	2.66598
742	353.21	60	21.19272
743	494.82	60	29.68932
744	354	60	21.24
745	218	60	13.08
747	744	60	44.64
748	41.7	60	2.50194
750	584	60	35.04
753	582	60	34.92
758	85.61	60	5.13642
761	404.97	60	24.29838
762	339.41	60	20.36484
763	26.26	60	1.57566
764	192.53	60	11.55204
765	482	60	28.92
766	538	60	32.28
767	554	60	33.24
769	454	60	27.24
771	32.76	60	1.96578
772	448	60	26.88
774	430	60	25.8
777	430	60	25.8
778	980	60	58.8
780	1200	60	72
781	1120	60	67.2
782	928	60	55.68
785	872	60	52.32
786	552	60	33.12
787	718	60	43.08
788	556	60	33.36
790	696	60	41.76
791	670	60	40.2
792	388	60	23.28
794	552	60	33.12
795	370	60	22.2
796	508	60	30.48
797	482	60	28.92
800	582	60	34.92
801	586	60	35.16
804	646	60	38.76
805	538	60	32.28
807	718	60	43.08
811	462	60	27.72
816	444	60	26.64
818	770	60	46.2
819	212	60	12.72
820	610	60	36.6
823	694	60	41.64
824	484	60	29.04
825	348	60	20.88
826	426	60	25.56
427	213.91	60	12.8343
428	269.27	60	16.15608
429	152.9	60	9.17424
430	107.76	60	6.46554
433	149.21	60	8.95242
434	116.57	60	6.99396
435	320.71	60	19.24272
437	163.09	60	9.78528
439	130.83	60	7.8498
440	262	60	15.72
446	415.8	60	24.94776
447	239.65	60	14.37912
448	89.74	60	5.38464
454	298.74	60	17.92458
460	340	60	20.4
461	298	60	17.88
462	466.71	60	28.0023
463	341.99	60	20.51946
465	310.13	60	18.60762
467	179.5	60	10.76982
468	144.64	60	8.6784
473	162.82	60	9.7689
474	306.45	60	18.387
605	127.74	60	7.66434
606	268.72	60	16.1232
609	233.97	60	14.03808
612	336.64	60	20.19858
615	255.17	60	15.31038
616	283.12	60	16.98696
617	484	60	29.04
618	102.1	60	6.12576
619	194.28	60	11.65698
620	119.26	60	7.15542
624	107.45	60	6.44724
626	368.37	60	22.1022
627	106.09	60	6.36558
628	236.8	60	14.2077
631	99.48	60	5.9685
633	95.01	60	5.70048
634	103.34	60	6.2001
635	109.99	60	6.59928
636	64.64	60	3.8781
637	87.75	60	5.26512
638	100.91	60	6.05466
639	88.95	60	5.33718
641	132.69	60	7.96158
643	204.28	60	12.25674
650	76.79	60	4.60728
651	168.02	60	10.0812
653	209.06	60	12.54336
655	418.67	60	25.12032
658	380.74	60	22.84446
661	205.38	60	12.32256
662	298.04	60	17.8824
664	591.32	60	35.47932
665	386.54	60	23.19234
666	366.54	60	21.9921
667	211.01	60	12.66084
668	305.35	60	18.32118
670	505.6	60	30.33582
678	473.56	60	28.41378
680	583.45	60	35.00706
682	376.9	60	22.61406
686	426.4	60	25.5837
687	442.71	60	26.56266
688	500.23	60	30.01392
690	555.19	60	33.31152
692	268.51	60	16.11084
694	284.53	60	17.07192
695	448	60	26.88
696	333.69	60	20.02128
697	455.07	60	27.30432
700	569.97	60	34.19796
703	314.46	60	18.8676
705	253.77	60	15.22632
708	280.86	60	16.85178
709	271.21	60	16.2723
712	185.48	60	11.12892
713	249.57	60	14.97444
714	416.3	60	24.97824
715	354.83	60	21.29004
717	37.29	60	2.23746
718	184.58	60	11.0745
827	95.29	60	5.71764
828	267.7	60	16.06188
829	256.28	60	15.37698
830	275.84	60	16.5504
831	312.1	60	18.72618
832	780	60	46.8
834	219.74	60	13.18446
835	90.31	60	5.41872
836	285.74	60	17.14422
837	462	60	27.72
841	231.66	60	13.8996
843	411.4	60	24.68382
848	397.33	60	23.83998
849	466	60	27.96
850	502	60	30.12
851	366.59	60	21.99516
852	648	60	38.88
853	37.03	60	2.22174
854	486	60	29.16
859	460	60	27.6
861	701	60	42.06
862	348.1	60	20.88582
866	402	60	24.12
868	191.16	60	11.46966
870	544	60	32.64
872	301.79	60	18.10752
873	478.29	60	28.69764
874	289.01	60	17.34066
875	542	60	32.52
878	29.34	60	1.76064
879	21.14	60	1.2681
881	544	60	32.64
882	195.73	60	11.74362
883	548	60	32.88
884	369.57	60	22.17396
885	318.24	60	19.09434
888	486	60	29.16
889	460	60	27.6
891	202.48	60	12.1488
892	220.65	60	13.23882
893	352	130	45.76
895	235.94	130	30.67259
899	30.94	60	1.85628
900	353.51	130	45.95578
901	238	130	30.94
902	502	130	65.26
903	50.78	130	6.60192
904	334	130	43.42
905	448.44	130	58.29658
911	334.39	130	43.4708
912	356.51	130	46.34635
913	397.44	130	51.6672
914	475.32	130	61.79121
915	478.27	130	62.17497
916	392.98	130	51.0875
918	1020	130	132.6
919	428.47	130	55.70074
920	375.92	130	48.86947
921	485.96	130	63.1747
922	468.72	130	60.93305
927	404.72	130	52.61334
929	443.53	130	57.65908
930	338.17	130	43.96197
931	339.01	130	44.07109
932	350.68	130	45.5878
933	389.93	130	50.69103
934	354.19	130	46.04418
935	457.94	130	59.53194
936	362.48	130	47.12214
938	389.07	130	50.57949
939	361.36	130	46.97729
943	332.7	130	43.25116
944	363.62	130	47.27042
946	442.77	130	57.55997
953	413.29	130	53.72718
954	344.66	130	44.80531
955	322.2	130	41.88639
956	336.58	130	43.75504
957	384.55	130	49.99129
958	436.97	130	56.8061
959	326.5	130	42.4451
960	357.26	130	46.44377
962	380.7	130	49.49116
963	410.2	130	53.32613
964	402.55	130	52.3315
965	484.12	130	62.93573
966	356.09	130	46.29136
968	413.37	130	53.73797
969	480.94	130	62.52277
970	351.21	130	45.65696
975	420.35	130	54.64524
976	446.57	130	58.05415
977	360.81	130	46.90517
980	406.76	130	52.87924
981	465.2	130	60.47574
982	433.07	130	56.29845
983	483.84	130	62.89907
985	454.13	130	59.03711
986	340.35	130	44.24519
987	474.46	130	61.67928
876	464	60	27.84

**Table A3:** Statistics of SNP detection and annotation results.

	Category	Number of SNPs
	Upstream	181,049
Exonic	Stopgain	437
Exonic	Stoploss	53
Exonic	Synonymous	129,104
Exonic	Non-synonymous	59,403
	Intronic	5,746,658
	Splicing	226
	Downstream	166,387
	Upstream/Downstream	9724
	Intergenic	6,350,604
	Ts	8,995,777
	Tv	3,648,686
	ts/tv	2.465
	Total	12,644,463

**Table A4:** Significant SNP classification annotation result statistics.

	Category	Number of SNPs
	Upstream	2
Exonic	Synonymous	1
Exonic	Non-synonymous	1
	Intronic	138
	Intergenic	76
	Total	218

**Table A5:** Information table of significant SNPs on sex chromosomes (top 20 *p*-values).

Chr	Num_snp	LOG10 (Peak *p*-Value)	Peak-Effect
Chr1	1	8.87361	exon, synonymous
ChrZ	3	8.28455	intronic(gene_DTWD2)
ChrZ	13	8.02251	intronic(gene_NRG1)
Chr1	3	7.87361	intergenic(/)
ChrZ	9	7.81761	intronic(gene_ANKDD1B)
Chr20	11	7.38612	intergenic(gene_EIF6(dist = 1613))
Chr20	3	7.29405	intronic(gene_FAM83C)
Chr4	1	7.00458	intergenic(/)
ChrZ	3	6.87857	intergenic(/)
Chr27	1	6.86258	intronic(gene_HAP1)
ChrZ	15	6.82981	intronic(gene_BNC2)
ChrZ	5	6.75517	intergenic(/)
Chr4	1	6.74306	intergenic(/)
Chr10	1	6.72638	intronic(gene_UACA)
ChrZ	1	6.69983	intergenic(/)
Chr39	1	6.68551	intronic(gene_SCYL1)
ChrZ	3	6.62974	intronic(gene_TRIM36)
Chr2	2	6.57298	intronic(gene_TRAPPC9)
ChrZ	2	6.55467	intergenic(/)
ChrZ	1	6.5532	intergenic(/)

**Table A6:** Information table of significant SNPs in A series (top 10 *p*-values).

chr	LOG10 (*p*-Value)	Peak-Effect
Chr28	5.54968	downstream(gene_ANKRD24)
Chr6	5.32531	downstream(gene_MCMBP)
Chr6	5.32531	downstream(gene_MCMBP)
Chr28	5.49501	downstream(gene_UQCR11)
Chr1	5.43904	intergenic(gene_BTG1(dist = 180,311), gene_PLEKHG7(dist = 2624))
Chr1	5.44633	intergenic(gene_DCN(dist = 309,120), gene_BTG1(dist = 34,815))
Chr1	5.38578	intergenic(gene_LOC100858183(dist = 190,260), gene_EPYC(dist = 90,243))
Chr1	5.36285	intergenic(gene_LOC100858183(dist = 194,698), gene_EPYC(dist = 85,805))
Chr1	5.36285	intergenic(gene_LOC100858183(dist = 198,500), gene_EPYC(dist = 82,003))
Chr1	5.67468	intergenic(gene_LOC100858183(dist = 201,260), gene_EPYC(dist = 79,243))

**Table A7:** Differential gene table.

Gene	Organization	*p*-Value	ALV Expression	Control Expression
CDH11	Kidney	0.012442	357.8311495	830.4326925
ANKH	Liver	0.002114	2278.261492	926.0824854
SLC5A1	Spleen	0.005622	47.73315057	129.0051109
